# A Novel Ex Vivo Bone Culture Model for Regulation of Collagen/Apatite Preferential Orientation by Mechanical Loading

**DOI:** 10.3390/ijms23137423

**Published:** 2022-07-04

**Authors:** Ryota Watanabe, Aira Matsugaki, Takuya Ishimoto, Ryosuke Ozasa, Takuya Matsumoto, Takayoshi Nakano

**Affiliations:** 1 Division of Materials and Manufacturing Science, Graduate School of Engineering, Osaka University, Suita 565-0871, Japan; ryota.watanabe@mat.eng.osaka-u.ac.jp (R.W.); matsugaki@mat.eng.osaka-u.ac.jp (A.M.); ishimoto@mat.eng.osaka-u.ac.jp (T.I.); ozasa@mat.eng.osaka-u.ac.jp (R.O.); 2 Teijin Nakashima Medical Co., Ltd., 688-1 Joto-Kitagata, Higashi-ku, Okayama 709-0625, Japan; 3 Department of Biomaterials, Graduate School of Medicine, Dentistry and Pharmaceutical Sciences, Okayama University, 2-5-1, Shikata-cho, Kita-ku, Okayama 700-8558, Japan; tmatsu@md.okayama-u.ac.jp

**Keywords:** collagen/apatite orientation, ex vivo, mechanical loading, osteocyte morphology, endochondral ossification

## Abstract

The anisotropic microstructure of bone, composed of collagen fibers and biological apatite crystallites, is an important determinant of its mechanical properties. Recent studies have revealed that the preferential orientation of collagen/apatite composites is closely related to the direction and magnitude of in vivo principal stress. However, the mechanism of alteration in the collagen/apatite microstructure to adapt to the mechanical environment remains unclear. In this study, we established a novel ex vivo bone culture system using embryonic mouse femurs, which enabled artificial control of the mechanical environment. The mineralized femur length significantly increased following cultivation; uniaxial mechanical loading promoted chondrocyte hypertrophy in the growth plates of embryonic mouse femurs. Compressive mechanical loading using the ex vivo bone culture system induced a higher anisotropic microstructure than that observed in the unloaded femur. Osteocytes in the anisotropic bone microstructure were elongated and aligned along the long axis of the femur, which corresponded to the principal loading direction. The ex vivo uniaxial mechanical loading successfully induced the formation of an oriented collagen/apatite microstructure via osteocyte mechano-sensation in a manner quite similar to the in vivo environment.

## 1. Introduction

Bone is an organ system that performs various functions, including weight support, movement support, and mineral storage. Japan is a super-aging society with an elderly population that exceeds 25% of the total population [1]. The overall risk of fractures may therefore increase owing to the deterioration of bone mechanical function with aging. Therefore, there is a need to establish diagnostic methods that can accurately assess bone strength and to develop therapeutic methods that can restore bone mechanical properties at an early stage.

Bone tissue has a hierarchical and anisotropic microstructure comprising collagen fibers and apatite crystals [2], which undergo mechanical adaptation by altering their density and structure depending on the surrounding stress environment [3,4,5,6]. The direction and degree of the anisotropic microstructure composed of collagen fibers and apatite crystals vary with the stress distribution at each in vivo site. Moreover, it tends to be preferentially oriented along the longitudinal axis of the bone, which is the direction of loading in long bones [7]. The preferential orientation of collagen/apatite has been shown to dictate the mechanical function of bone more than bone mineral density [8]. Bones with a strongly oriented collagen/apatite structure exhibit high anisotropy in Young’s modulus [9], yield stress [10], and ultimate stress [9,11]. On the contrary, bones with poorly oriented collagen/apatite structures, including regenerating bones [12,13], are found to be isotropic and exhibit a low Young’s modulus [14]. Recent studies have shown that collagen/apatite preferential orientation is an indicator of bone health [15]; it is thus important to elucidate the factors controlling collagen/apatite orientation to establish innovative diagnostic and therapeutic strategies for strengthening bones. The degree and direction of collagen/apatite preferential orientation are affected when the mechanical environment around the bone is altered by prosthetic replacement [16] and artificial loading [17]. However, the mechanism underlying the formation of the oriented collagen/apatite structure, based on changes in the mechanical environment, is not understood.

In vivo stress distributions are altered in a complex and dynamic manner, including mechanical stimuli from the muscles surrounding the bone [18] and changes in the stress distributions associated with walking [19]. Through in vitro cell experiments, we attempted to control the stress environment around mouse osteoblasts and revealed osteoblast alignment and the consequent production of the anisotropic bone matrix by continuous stretch stimulation [20]. However, cell-based experimental systems have limitations, including the two-dimensional environment surrounding the bone cells, which is far from their three-dimensional spatial distribution in living tissues. In this study, we developed an ex vivo organ culture model that mimics living bone dynamics in a three-dimensional culture system equipped with a controllable device for mechanical conditions. We aimed to establish an ex vivo bone culture model capable of controlling loading conditions and investigated whether it could induce similar changes in the preferential orientation of collagen/apatite as those observed in vivo.

## 2. Results

### 2.1. Establishment of an Ex Vivo Bone Culture Model

**Figure 1:**
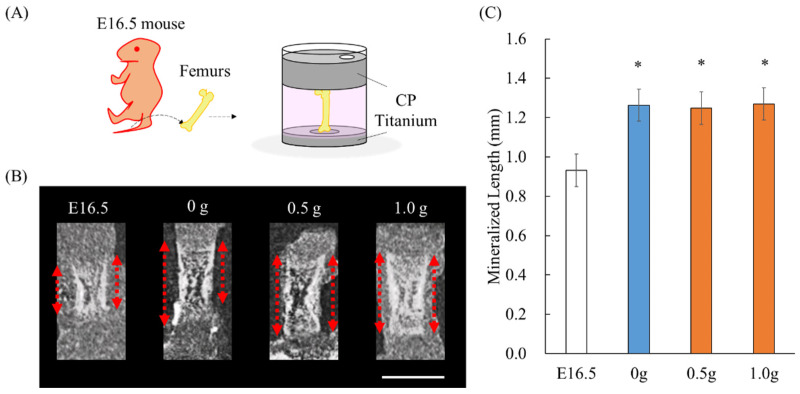
Ex vivo bone culture model and micro-CT analysis: (**A**) Schematic illustration of ex vivo model, (**B**) longitudinal images by micro-CT scanning (red dot line: mineralized length), (**C**) mineralized length of the femur before and after cultivation. Scale bar: 1 mm. * *p* < 0.05 (vs. E16.5).

Micro-computed tomography (Micro-CT) cross-sectional images showed that the mineralized area of the bone after cultivation in the ex vivo bone culture system with or without mechanical loading was enlarged (Figure 1B). Figure 1C shows the length of the mineralized area evaluated from micro-CT cross-sectional images. The length of the mineralized segment in all cultured groups significantly increased as compared with that of embryonic 16.5-day-old (E16.5) femurs (0 g: *p* = 0.012, 0.5 g: *p* = 0.016, 1.0 g: *p* = 0.011, where “g” means gram).

**Figure 2:**
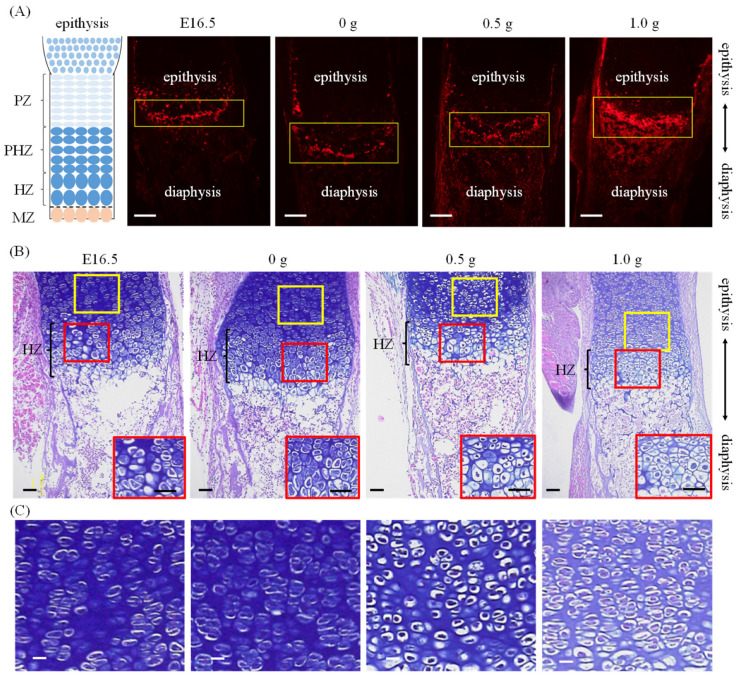
Immunofluorescence and histological images: (**A**) Schematic illustration of growth plate structure and immunohistochemical images of the metaphyseal of femurs. The boxed regions indicate hypertrophic zones or mineralized areas. Red: osteopontin, Scale bars: 100 µm, PZ: Proliferative zone, PHZ: Pre-hypertrophic zone, HZ: Hypertrophic zone, MZ: Mineralized zone. (**B**) Histological images stained with Alcian blue and HE around the metaphyseal of femurs. The insets show the magnified HZs of each femur (Red rectangular area). Scale bars: 50 mm. (**C**) Magnified images of the yellow boxed areas on the upper side are shown.

Immunostaining showed no major changes in the expression of osteopontin in cultured femurs loaded at 0 g and 0.5 g compared to that of E16.5 (Figure 2A). In contrast, osteopontin expression was markedly elevated when femurs were loaded at 1.0 g. Alcian blue/HE staining confirmed the hypertrophic zones (HZs) in both bones before and after culture (Figure 2B). At E16.5 and 0 g, almost no pre-hypertrophic zones (PHZs) were observed on the epiphyseal side of the HZs, whereas the PHZs were visible at 0.5 g and 1.0 g.

### 2.2. Preferential Apatite Orientation along Femur Long Axis with/without Mechanical Loading

**Figure 3:**
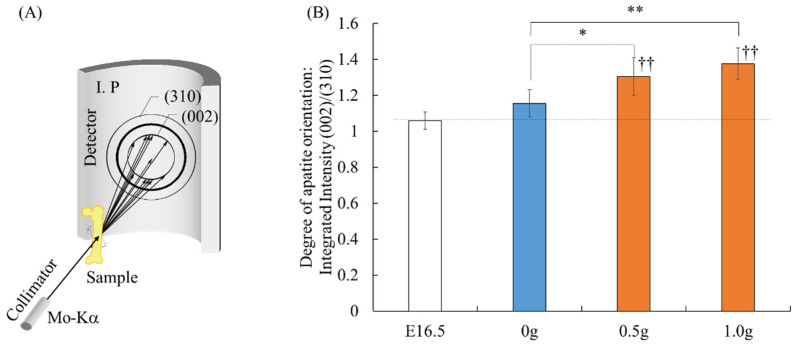
Analysis of apatite orientation: (**A**) Schematic illustration of the apatite orientation measurement using transmission micro-beam XRD method. The preferential orientation of the *c*-axis of apatite crystals was analyzed with the integrated intensity ratio of (002)/(310). (**B**) Quantitative analysis of apatite orientation along the longitudinal direction of the bone before cultivation (E16.5) and after 7 days of cultivation. * *p* < 0.05, ** *p* < 0.01. †† *p* < 0.01 vs. E16.5.

Figure 3 shows the *c*-axis orientation of the preferential apatite in the diaphysis, quantitatively analyzed using the micro-beam X-ray diffractometer (μXRD) system. The apatite orientation was found to be significantly higher after uniaxial mechanical loading than before culture (0.5 g; *p* = 0.0003, 1.0 g; *p* = 0.00002). In cultured femurs with no mechanical loading (0 g), the apatite orientation showed no significant difference. Femurs loaded with 0.5 g and 1.0 g femurs showed a significantly higher degree of apatite orientation than the non-loaded femurs did (0.5 g; *p* = 0.020, 1.0 g; *p* = 0.0009).

**Figure 4:**
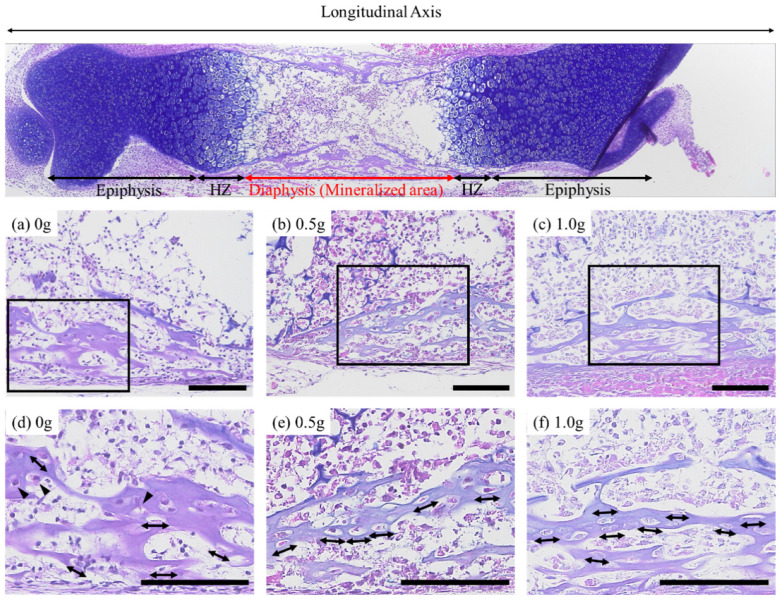
Histological image stained with Alcian Blue and HE of entire bone and the magnified images in the diaphysis of (**a**) 0 g, (**b**) 0.5 g and (**c**) 1.0 g, and (**d**–**f**) magnified images of the boxed regions. Arrowheads indicate spherical osteocytes. Arrows indicate the elongation of osteocytes. Scale bars: 100 mm.

Figure 4 shows Alcian blue/HE staining images of the femurs after seven days of cultivation. In the non-loaded bone, osteocytes showed a spherical or elongated morphology, depending on the locations. On the contrary, in the 0.5 g and 1.0 g loaded bones, the osteocytes exhibited an anisotropic shape with a preferential alignment in the loading direction.

## 3. Discussion

In this study, we aimed to establish an ex vivo bone culture model that could be used to regulate the preferential orientation of collagen/apatite by controlling artificial loading. We evaluated changes in the endochondral ossification and apatite orientation before and after culture, as well as in the osteocyte morphology and alignment in the bone matrix.

During embryonic development, bone formation appears to occur via endochondral ossification, where bone growth proceeds along its long axis. Endochondral ossification progresses in growth plates through the differentiation of resting chondrocytes into proliferative, hypertrophic, or mineralized chondrocytes, while producing a specific extracellular matrix containing type II collagen [21,22]. Micro-CT images of femurs from embryonic mice revealed a progressive mineralization with increasing days of embryonic life, supporting the occurrence of endochondral ossification in embryonic bones [23]. In this study, we performed micro-CT imaging to clarify whether the bone cultured in our ex vivo model grew via endochondral ossification. We found that the mineralized length of the bone in our model with or without mechanical loading expanded in the long direction (Figure 1B,C). Chondrocyte differentiation was evaluated using histological staining and immunostaining. The expression of osteopontin, a protein expressed in HZs and mineralized zones [24,25,26], was altered depending on the loading force, and an increased expression with a higher loading force was observed in the metaphyseal region (Figure 2A). This result indicated that differentiation into HZs and calcification were promoted by moderate loading. Additionally, the sections stained with Alcian blue and HE, which distinguish between cartilage and bone tissue, respectively, showed accelerated hypertrophy of chondrocytes owing to loading (Figure 2B,C). The suppression of chondrocyte hypertrophy has been reported [27,28,29] upon application of static uniaxial loading to the bone using postnatally grown bones. These differences may be associated with the difference in loading histories of the embryonic bones used in the present study. Progressive mineralization in embryonic bone with static uniaxial loads [30] suggested that chondrocyte hypertrophy was not inhibited. Therefore, the ex vivo bone culture model established here is a culture system that can induce the progression of endochondral ossification based on the control of load history. However, the lack of vascularization in ex vivo experiments should be considered. Since vascularization is essential for the mineralization of the developing bone, the lack of vascularization might induce delayed mineralization. Our ex vivo cultured femur showed a mineralized length lower than that reported in an in vivo analysis of growth from E16.5 to postnatal-6 day [31]. Additionally, angiogenesis plays an important role in bone development and regeneration [32] in relation to mechanical loading [33]. The effect of mechanical loading on angiogenesis needs further exploration.

In this study, changes in apatite orientation by manipulating the loading history of embryonic femurs using our ex vivo bone culture model were evaluated (Figure 3B). The results showed that the apatite orientation of femurs loaded with uniaxial forces was significantly higher than that of the unloaded femurs. We previously reported that the arrangement of the collagen/apatite microstructure was determined by controlling the artificial loading to the bone [17,34,35]. The findings of the present study are consistent with the changes in apatite orientation observed in these in vivo studies. Considering the important relationship between mechanical performance and bone microstructure, the highly aligned bone cultured under loading conditions could realize the superior mechanical functions. Regarding the principal in vivo stress applied along the longitudinal direction, we focused on the effects of longitudinal loading on the bone microstructure. Lateral loading is also important because the excess alignment of apatite in bone diseases, such as osteoporosis, shows a high fracture risk against lateral loading [36].

The change in apatite orientation based on mechanical loading/unloading was attributed to functional adaptation via the mechano-sensitivity of the osteocytes. Osteocytes form lacuna-canalicular networks in the bone matrix and regulate the bone morphology and volume suitable for the surrounding mechanical environment by detecting the interstitial fluid flow in the canaliculi caused by external mechanical loading [37,38,39]. It is also believed that osteocytes are efficiently sensitive to stress fields; they alter their morphology and that of the lacuna-canalicular network in response to external stress [40,41]. It has been reported that osteocytes in healthy bones are elongated and aligned along the long axis of the bones loaded with anisotropic mechanical loads, but osteocytes are spherical and randomly aligned under unloading conditions [17]. Moreover, a recent study revealed that the morphology and alignment of osteocytes are closely related to apatite orientation [42]. In bones with a high degree of apatite orientation, the elongation and alignment of osteocytes parallel to the loading direction were observed. In other words, the functional adaptation of apatite orientation via osteocytes’ mechano-sensation can be observed through their morphology and alignment. In this study, osteocytes in the bone matrix were observed in the Alcian blue/HE-stained sections (Figure 4). Spherical osteocytes and osteocytes elongated in the long-axis direction were detected in bones without mechanical loads. However, osteocytes were scattered across various locations, and almost no alignment was noted. In contrast, osteocytes elongated in the load-bearing direction were localized in the bone tissues loaded with anisotropic mechanical stimuli and were confirmed to be aligned in comparison with those in the unloaded bones. This observation indicates that osteocytes are susceptible to anisotropic loading and form aligned lacuna–canalicular networks, where the canaliculus is extended in the direction perpendicular to the loading direction. In other words, our results indicated that the application of artificial mechanical loading using our ex vivo bone culture model induced bone formation with a preferentially oriented collagen/apatite microstructure through the mechano-sensitivity of osteocytes. Our previous report clarified the strict correlation between the anisotropy of the bone microstructure and the lacuno-canalicular network of osteocytes [42]. Elongated osteocytes show an efficient sensing of uniaxial mechanical loading by modifying the preferential orientation of collagen/apatite, which may strengthen bone along the loading direction. It is therefore clear that the ex vivo bone culture model developed in this study is a system that enables the formation of collagen/apatite-oriented bones by a biological mechanism similar to that observed in vivo.

Mechanical adaptation of the bone has attracted considerable attention in the past few decades. In recent years, it has become clear that not only bone volume and shape but also bone quality, such as collagen/apatite orientation, undergo a functional adaptation to external mechanical stimuli [3,4,5,6,13,14]. In this study, we established an ex vivo bone culture model that enabled the artificial regulation of static uniaxial loading and successfully induced the mechanical adaptation of the bone microstructure via the mechano-sensitivity of osteocytes. However, in actual life, both static and dynamic loads are applied to the bone, and the adaptive responses to dynamic loads play important roles in biological function [43,44]. In particular, our recent findings suggest that the dynamic loading condition realizes the anisotropic bone matrix microstructure by controlling the osteoblast arrangement [45]. An expanded ex vivo bone culture model that also enables the application and regulation of dynamic loads is required to further clarify the mechanism of collagen/apatite organization associated with mechanical loading.

## 4. Materials and Methods

### 4.1. An Ex Vivo Bone Culture Model

E16.5 mice (ICR; Japan SLC, Shizuoka, Japan) were anesthetized, and femurs were removed under a light microscope. Referring to previously reported ex vivo bone culture models [34,35], embryonic femurs were cultured in 96-well plates (IWAKI, Tokyo, Japan) and commercially pure (CP) titanium specimens (weights: 0.5 or 1.0 g) for 7 days at 37 °C in 5% CO_2_. The bone was fractured by a 1.5 g load. Therefore, we chose 1 g as the largest weight. The femurs were placed between the CP titanium specimens that possessed hollows for holding bones, to perform uniaxial static loading along the long axis of the bone (Figure 1A). The load intensity corresponded to the weight of the CP Ti specimens, which were continuously put onto the femur during cultivation. As a control, a floating culture without a pure titanium specimen was prepared (0 g). The α-modified Eagle’s medium (α-MEM; GIBCO) containing 10% fetal bovine serum (GIBCO, Grand Island, NY, USA), 50 μg/mL ascorbic acid (Sigma, St. Louis, MO, USA), 10 mM β-glycerophosphate (Tokyo Kasei, Tokyo, Japan), and 50 nM dexamethasone (MP Bioscience, Solon, OH, USA) was used as the culture medium. After seven days of cultivation, femurs were fixed in 10% neutral buffered formaldehyde. The medium was not changed during cultivation; however, half the volume of fresh medium was added at the midpoint of cultivation. All animal experiments were approved by the Ethics Committee for Animal Experiments at Osaka University. The application sheet for animal experiments, including the experimental contents of anesthesia protocol, number of animals, and categories of invasiveness, was approved by the Ethics Committee (approval number: 27-2-1).

### 4.2. Bone Morphology Analysis

Femurs before and after culture were scanned using micro-computed tomography (SMX-100CT; Shimadzu, Kyoto, Japan). Micro-CT scanning was performed at an X-ray energy setting of 39 kV and a current of 90 mA. The length of the mineralized region in the femoral shaft was measured from micro-CT cross-sectional images using ImageJ software (National Institute of Health, Bethesda, MD, USA).

### 4.3. Analysis of Apatite Orientation

The degree of apatite orientation in the femoral diaphysis was assessed using a micro-beam X-ray diffractometer system (R-Axis BQ, Rigaku, Tokyo, Japan) equipped with a transmission optical system. The incident beam (Mo-Kα) was irradiated perpendicular to the long axis of the bone at a tube voltage of 50 kV and a tube current of 90 mA. The preferential degree of the apatite *c*-axis orientation was determined as the relative intensity ratio of the (002) diffraction peak to the (310) peak in the X-ray profile [7,12].

### 4.4. Histological Analysis

The cultured femurs were fixed in 10% neutral buffered formaldehyde and decalcified by immersion in 10% formic acid for 7 days. The tissue specimens were dehydrated using a graded ethanol series (50% → 70% → 100%) and embedded in paraffin. Additionally, 5 μm thick sections were obtained from paraffin-embedded specimens using a microtome. The sections were subjected to deparaffinization by immersion in a lemozole and ethanol series (100% → 90% → 70%) and then stained with Alcian blue and hematoxylin/eosin (HE). The stained sections were observed under a bright field using a fluorescence microscope (Biozero, Keyence, Osaka, Japan).

### 4.5. Immunohistochemistry

Approximately 5 μm thick sections were prepared using the same method as for the histological sections. Normal goat serum (Invitrogen) and phosphate-buffered saline-trypsin (PBST) were used to block non-specific binding sites. The primary reaction was subsequently performed at 4 °C for 12 h using a primary antibody (anti-osteopontin, Rockland). After washing with PBST, the secondary antibody was applied for 60 min at room temperature. After thorough washing with PBST, the samples were sealed and observed using a fluorescence microscope (Biozero).

### 4.6. Statistical Analysis

Data obtained in this study are expressed as the mean ± standard deviation (SD). EZR software [46] was used for statistical analyses. Statistical significance between groups was tested using Tukey’s test, and the level of statistical significance was set at *p* < 0.05.

## 5. Conclusions

In this study, we established an ex vivo bone culture model that enabled the regulation of mechanical loading and evaluated the progression of endochondral ossification and the degree of apatite orientation therein. We succeeded in promoting endochondral ossification and controlling apatite orientation. Furthermore, our results indicate that the changes in apatite orientation observed in our ex vivo bone culture model were based on the regulation of the mechano-sensitivity of osteocytes, a mechanism that occurs in vivo. Based on these findings, we succeeded in developing an ex vivo bone culture system that induces the formation of collagen/apatite-oriented bone based on a mechanism similar to that observed in vivo.

## Data Availability

The data presented in this study are available upon reasonable request from the corresponding author.
